# Ointments and Oleates

**Published:** 1891-01

**Authors:** 


OINTMENTS AND OLEATES; Especially in Diseases of the Skin By John V. Shoemaker, A. M., M. D.; Professor of Materia Medical, and of Diseases of the Skin in the Medico-Chirurgical College of Philadelphia, etc. Second Edition, revised and enlarged ; 12mo., Cloth, 298 pages ; price, $1.50. Philadelphia and London : F. A. Davis.

We are pleased to have this volume in our library. The subject matter is of decided interest, and the manner with which the author has treated the subject, is very practical indeed, and shows evidence of great care in the arrangement. The mode of preparation is given, and the therapeutical application described under each preparation. Professor Shoemaker is an authority upon skin diseases, and is so recognized by the profession both at home and abroad, and what he has to say about "Ointments and Oleates" will certainly attract the attention of all who limit their practice to skin diseases. The work is excellent in all of its appointments.

